# The RIN: an RNA integrity number for assigning integrity values to RNA measurements

**DOI:** 10.1186/1471-2199-7-3

**Published:** 2006-01-31

**Authors:** Andreas Schroeder, Odilo Mueller, Susanne Stocker, Ruediger Salowsky, Michael Leiber, Marcus Gassmann, Samar Lightfoot, Wolfram Menzel, Martin Granzow, Thomas Ragg

**Affiliations:** 1 Agilent Technologies, Hewlett-Packard-Strasse 8, 76337 Waldbronn, Germany.; 2 Agilent Technologies, 5301 Stevens Creek Blvd., Santa Clara, CA 95051, USA; 3 ABP, Schiphol Boulevard 239, 1118 BH Schiphol. The Netherlands.; 4 Roche Diagnostics GmbH, Nonnenwald 2, 82372 Penzberg, Germany.; 5 Universität Karlsruhe, ILKD, Am Fasanengarten 5, 76131 Karlsruhe, Germany.; 6 quantiom bioinformatics GmbH & Co. KG, Ringstrasse 61, 76356 Weingarten, Germany.

## Abstract

**Background:**

The integrity of RNA molecules is of paramount importance for experiments that try to reflect the snapshot of gene expression at the moment of RNA extraction. Until recently, there has been no reliable standard for estimating the integrity of RNA samples and the ratio of 28S:18S ribosomal RNA, the common measure for this purpose, has been shown to be inconsistent. The advent of microcapillary electrophoretic RNA separation provides the basis for an automated high-throughput approach, in order to estimate the integrity of RNA samples in an unambiguous way.

**Methods:**

A method is introduced that automatically selects features from signal measurements and constructs regression models based on a Bayesian learning technique. Feature spaces of different dimensionality are compared in the Bayesian framework, which allows selecting a final feature combination corresponding to models with high posterior probability.

**Results:**

This approach is applied to a large collection of electrophoretic RNA measurements recorded with an Agilent 2100 bioanalyzer to extract an algorithm that describes RNA integrity. The resulting algorithm is a user-independent, automated and reliable procedure for standardization of RNA quality control that allows the calculation of an RNA integrity number (RIN).

**Conclusion:**

Our results show the importance of taking characteristics of several regions of the recorded electropherogram into account in order to get a robust and reliable prediction of RNA integrity, especially if compared to traditional methods.

## Background

The RNA molecule plays a critical role in transferring information encoded in the genome (DNA) to the many different forms of proteins. After extracting RNA from cells by various methods, scientists are provided with a direct measure of cellular activity using gene expression measurement techniques. Among these, real-time PCR and DNA microarrays are the most widely used techniques.

### Importance of integrity

RNA is a thermodynamically stable molecule, which is, however, rapidly digested in the presence of the nearly ubiquitous RNase enzymes. As a result, shorter fragments of RNA commonly occur in a sample, which can potentially compromise results of downstream applications [1,2]. In order to evaluate the degree of degradation, electrophoretic methods have been applied that separate the samples according to the size of the comprised molecules.

Historically, RNA integrity is evaluated using agarose gel electrophoresis stained with ethidium bromide, which produces a certain banding pattern [3]. Typically, gel images show two bands comprising the 28S and 18S ribosomal RNA (rRNA) species and other bands where smaller RNA species are located. RNA is considered of high quality when the ratio of 28S:18S bands is about 2.0 and higher. Since this approach relies on human interpretation of gel images, it is subjective, hardly comparable from one lab to another, and the resulting data cannot be processed digitally.

### Towards an automated approach

In 1999, the Agilent 2100 bioanalyzer was introduced for the separation of DNA, RNA, and protein samples. It has since become a mainstream technique for the analysis of RNA samples. The bioanalyzer is an automated bio-analytical device using microfluidics technology that provides eletrophoretic separations in an automated and reproducible manner [4]. Tiny amounts of RNA samples are separated in the channels of the microfabricated chips according to their molecular weight and subsequently detected via laser-induced fluorescence detection. The result is visualized as an electropherogram where the amount of measured fluorescence correlates with the amount of RNA of a given size.

Since data are produced in a digital format, they can be easily re-processed to allow additional calculations based on the acquired raw data. When first released, the *Biosizing Software *calculated the ratio of the two ribosomal bands, following the commonly used approach for RNA integrity assessment. However, although assessing RNA quality with the bioanalyzer proves to be far superior to the slab gel based approach, the usage of ribosomal ratio for RNA quality assessment has several shortcomings. In many cases, ribosomal ratios showed only weak correlation with RNA integrity [2,5] and more in-depth analysis of the electrophoretic traces requires expert knowledge and is of a subjective nature.

**Figure 1:**
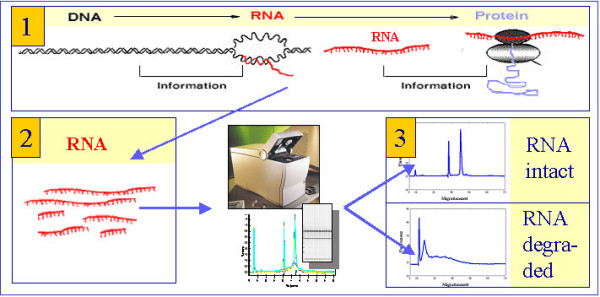
**Application environment**. (1) Role of RNA in gene expression and protein production, (2) extracted RNA molecules, measurement of RNA sizes applying Agilents 2100 bioanalyzer and (3) assignment of integrity categories to RNA samples. In the sample, RNA molecules of different sizes occur, which is measured by the 2100 bioanalyzer. The distinction with regard to integrity is based on this size distribution in each sample.

To provide a tool for standardization of RNA quality control, a user-independent, automated and reliable procedure was developed. In this study we present a software algorithm that allows the calculation of an RNA Integrity Number (RIN). The algorithm was developed using methods from information theory to rank features according to their information content and using a Bayesian approach to train and select a prediction model on the basis of artificial neural networks. An overview of the application scenario is summarized schematically in figure 1.

### Techniques for measuring RNA integrity

**Figure 2:**
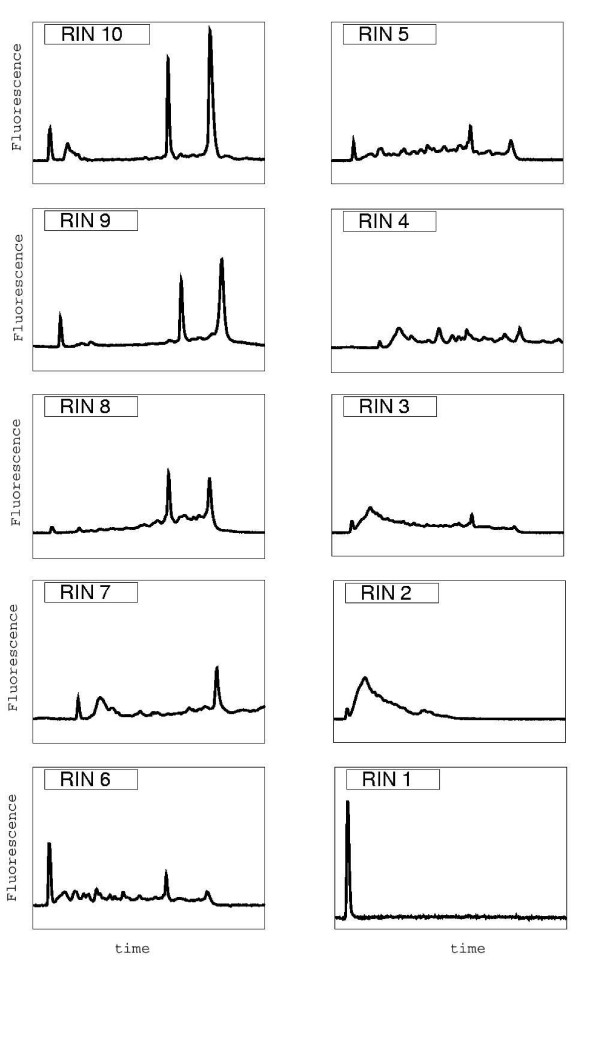
**RNA integrity categories**. The figure shows typical representatives of the ten integrity categories. RIN values range from 10 (intact) to 1 (totally degraded). The gradual degradation of rRNA is reflected by a continuous shift towards shorter fragment sizes.

For microcapillary electrophoresis, the Agilent 2100 bioanalyzer was used in conjunction with the RNA 6000 Nano and the RNA 6000 Pico LabChip kits. This bio-analytical device is based on a combination of microfluidic chips, voltage-induced size separation in gel filled channels and laser-induced fluorescence (LIF) detection on a miniaturized scale [4]. Twelve samples can be processed sequentially while consuming only very small amounts of each sample. RNA molecules are stained with an intercalating dye and detected by means of LIF. Data are archived automatically and available as electropherograms, gel-like images, as well as in tabular format. Figure 2 shows example electropherograms of different RNA samples displaying varying RNA integrity levels. Visual inspection of the electrophoretic traces reveals that progressing degradation entails a decrease of signal intensities for the two ribosomal bands in conjunction with an increase of shorter fragments, i.e. an elevated baseline between the two ribosomal bands and below the 18S band.

### Integrity measures for RNA

The degradation process of RNA is only partly understood since it is dependent on the type of RNase that is present and is often combined with fragmentation processes. Also, the quality of RNA in a given experiment can vary extensively from one extraction to another and needs to be under constant surveillance. Using precise analytical instrumentation such as the Agilent 2100 bioanalyzer, human experts are capable of distinguishing RNA samples of different quality by examining electrophoretic traces and assigning integrity values or integrity categories [2]. Note that the degradation of RNA is a continuous process, which implies that there are no natural integrity categories.

In this situation, machine learning methods can be applied to data from experimental measurements to learn an implicit model of RNA integrity in order to achieve a user-independent method for assigning integrity values. That is, the algorithm extracts the relationship between characteristics of the measurement, or features, and the expert-assigned categories.

Traditionally, a simple model evaluating the 28S to 18S rRNA ratio has been used as a criterion for RNA quality. If not restricted to very specific RNA types this model has been shown to be insufficient for a general RNA integrity prediction [2,5]. An adaptive approach can be applied to solve this prediction task when based on a large collection of samples. In general, an approach applied to this kind of task comprises at least the following basic steps, which are described in more detail below in the method section:

1. Data labeling and preprocessing

2. Extracting features from the electropherogram

3. Selecting an appropriate feature combination

4. Training prediction models and selecting the most promising model

## Results

### Sample preparation and data basis

Total RNA was obtained from various tissues and different organisms mainly human, rat, and mouse. All samples were analyzed on the Agilent 2100 bioanalyzer. For the development of the algorithm a large set of data files was kindly provided by the Resource Center for Genome Research [6] as well as by Agilent. The overall number of samples in the database totals 1208. About 30% of the samples are of known origin from human, mouse and rat extracted from liver, kidney, colon, spleen, brain, heart and placenta. The origin of the remaining samples was not traceable, but is known to be of mammalian cells or cell lines. For the development of the algorithm, it was important to include samples of all degradation stages into the database. The final data set included many intact as well as almost completely degraded RNA samples (cf. table 1 for the distribution of samples). Partially degraded RNA samples were less common but still sufficient in number. Furthermore, the data set comprised different sample concentrations and different extraction methods. To some extent anomalies were found in the data set as well. This provided a realistic collection of input data containing a representative basis for all stages of RNA degradation.

Applying our method described below to the data basis yields a sorted list of features, which was used to construct feature spaces for training regression models. Furthermore, results are given for models based on features proposed in the literature. Finally, we show the correlation of the RIN with the outcome of real-time PCR experiments.

### Feature selection

The total RNA ratio was selected as first feature covering 79% of the entropy of the categorical values. The next two features contribute information about the 28S-region: 28S peak height and the 28S area ratio.

The fourth feature compares the 18S and 28S area to the area of the fast region. Feature 5 is the value of a linear regression at the end point of the fast region, whereas the next feature reflects the amount of detected fragments in the fast region. Then, the presence or absence of the 18S peak is selected, which enables the model to distinguish between weaker and stronger degradation. The last feature gives the relation of the overall mean value to the median value. Since the mean value is sensitive to large peaks it carries information about totally degraded RNA or about abnormalities like spikes. Table 2 summarizes the results of the feature selection process. An interpretation of these features from a biological point of view is given in the discussion and an overview of all features is given in the additional files [see Additional file 2].

### Model training

**Figure 3:**
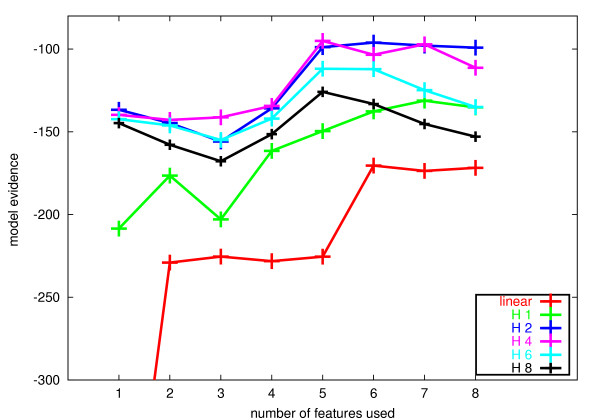
**Evidence-based model selection**. Dependency of model evidence on a logarithmic scale from the number of features used and from the degree of non-linearity in the hidden layer. The values are average values over a 10-fold cross-validation procedure. The highest evidence is reached for models with 5 to 7 input features and 2 to 5 hidden neurons respectively. All these models have a low generalization error below 0.25.

Based on the sorted list of features we trained neural networks as regression models and systematically increased the number of hidden neurons from 0 to 8, until the model evidence decreased clearly. Furthermore, we varied the feature space as described in the previous section. We observed maximal model evidence using 5 to 7 features with 2 to 5 neurons in the hidden layer. The values are averaged over the results of a 10-fold cross-validation procedure (Fig. 3).

**Figure 4:**
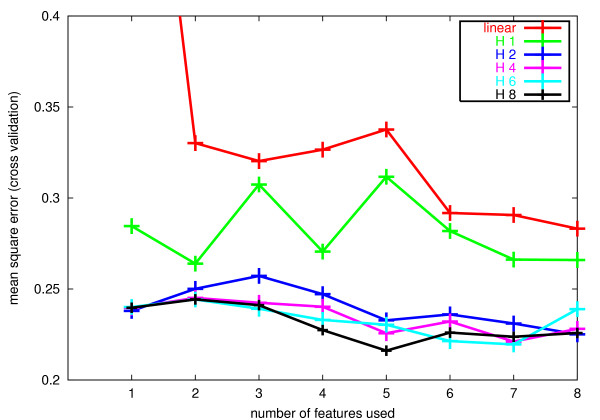
**Generalization errors**. Dependency of the generalization error of the model from the number of features used and from the degree of non-linearity in the hidden layer. The values are average values over a 10-fold cross-validation procedure. Models with highest evidence (5 to 7 input features and 2 to 5 hidden neurons respectively) have a low generalization error below 0.25.

As expected, the model evidence is strongly negatively correlated with the generalization error (*ρ *= -0.93), which shows that the model evidence is a sensible model selection criterion (Fig. 3 and 4). We selected the topology using 5 features and 4 hidden neurons as the most probable model and performed the final training on the whole training data set. The log value for the evidence of the final model was slightly higher compared to the values during cross validation (-74 vs. -100), whereas the generalization error was stable (MSE of 0.26). The cross validation error was observed to be a good estimate for the generalization error on the test data.

The feature selection procedure provides in each step the local optimal additional feature, which will not necessarily lead to the globally best combination. In the later iteration steps, several candidate features provide the same gain in information about the target and there is some randomness in the final selection. Explorative searching for the best combination is intractable because of the computational costs of the combinatorial search. In an additional, manual optimization step application knowledge was used to substitute some features by plausible alternatives. Feature 3 and 4 were replaced by the area ratio in the fast region (fast.area.ratio). Additionally the marker height was selected. In the normalized electropherogram, the marker height allows to detect strongly degraded samples, because it is the only part of the signal which differs from the background noise. This combination also has a relative MI value of 0.83, but the best model with 5 hidden neurons had a log value for the evidence of -42. It reaches a cross-validation error of 0.25 and a test error of 0.25, which is slightly better compared to the results from figure 4. Both models perform equally well, the later one was chosen for the final implementation in the expert software for the sake of simplicity.

Finally, we evaluated regression models for a subset of 400 samples on two different feature spaces: the 28S/18S ratio, and the feature computed by the degradometer software [1]. Table 3 shows that the RIN model is based on a feature space, with higher information content than the other two models. Model evidences indicate that using a single feature results in a lower posterior probability of the model. This is again consistent with the generalization performance of the models. The error of RIN model is forty times lower compared to 28S/18S-model and about twenty times lower compared the degradometer based model if all samples are considered.

If the samples that are labeled *BLACK *by the degradometer software are removed from the data set (*N *= 186, 42%), the relative MI value increases to 0.63, the evidence reaches a value of -121, whereas the cross validation error is at 0.60, which is still four times higher than for the RIN model.

### Model evaluation

If a model is supposed to extract a relationship from experimental data, it is helpful for the model evaluation to explore the data in the most important two dimensions, as well as to check for large error values which correspond to categorical misclassifications. Furthermore, the model prediction can be cross-checked against control parameters of follow-up experiments, like RT-PCR.

#### Visualization of decision boundaries

The 2D-visualization of total RNA ratio and 28S peak height shows, that we can clearly separate high integrity values from low integrity values. The categories form clusters in this space. However, as mentioned in the previous section, the borderline between categories is not sharp, which is due to the fact that degradation is a continuous process.

#### Categorical misclassifications

Simple features like the ribosomal ratio which cover only one aspect of the degradation process tend to have larger errors for certain groups of experiments. That is, they cannot distinguish very well between the categories. It is very useful to check, that only a few experiments are interchanged over more than one categorical border, that is, the model covers all aspects of the degradation process. Misclassifications are measured by Receiver Operating Characteristics (ROC, cf. [7]) for distinguishing electropherograms from different groups of categories, whereas the value of the area under the ROC-curve (AUC: area under the curve) is a balanced measure for the classification error.

We briefly and informally describe how a ROC curve is constructed. The electropherograms are sorted into a ordered list according to the integrity measure estimated by the model. For a fixed classification task, a ROC curve is constructed as exemplarily described in the following for the task of distinguishing electropherograms of category 10 from all other categories: walk through the sorted list in descending order. In each step check if the considered item is in category 10 or not according to the original expert label. If it is true, draw a line fragment in vertical direction, if it is false draw it in horizontal direction. Perfect separation of category 10 from the others would imply that the ROC curve shoots up vertically on the y-axis to the maximal value, before the first horizontal step is taken. Random assignment of electropherograms would result in a ROC curve that corresponds to a diagonal line from the origin to the right top corner. A ROC curve gives a balanced measure of the model performance by integrating over all possible classification borders. Each border corresponds to a specific ratio of sensitivity to specifity, i.e. a specific point on the ROC curve.

**Figure 5:**
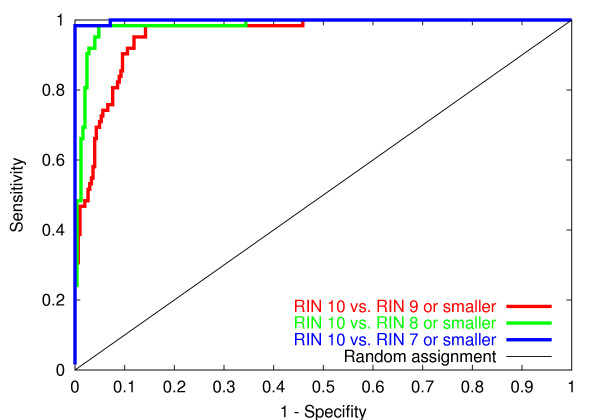
**Receiver operating characteristics of categorical misclassifications**. The figure shows the Receiver Operating Characteristics for distinguishing electropherograms of category 10 against the set union of other categories. The area under the curve (AUC) gives a measure of classification performance. Random assignment is equal to an area of 0.5, whereas perfect assignment is equal to an area of 1.0. Only few experiments are exchanged over more than one categorical border.

Several electropherograms are interchanged between the adjacent categories 9 and 10 (AUC 0.96), which is natural due to the noise in label assignment step. Very rarely are assignments from electropherograms from category 10 to category 8 or less (AUC 0.98). Only 1 electropherogram is interchanged between category 7 and category 10 (AUC 0.999, cf fig. 5).

Computing the AUC value for all other sensible groups of categories shows that categorical misclassifications are seldom observed. The average AUC-value is 98.7 with a standard deviation of 1.4. Table 4 summarizes the categorical errors over all possible sets of experiments.

### Correlation with the outcome of experiments

Correlation of RIN values with downstream experiments is of critical importance. On the one hand, a good correlation will demonstrate the validity of this approach. On the other hand, it allows determination of threshold values in order to get meaningful downstream data. For two-color microarray experiments, this could mean for example that the two input samples should not differ by more than a given number of RIN classes, while the lowest acceptable RIN can be determined as well.

**Figure 6:**
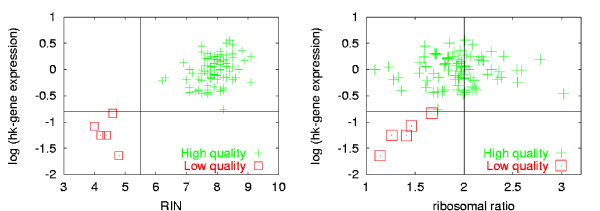
**Correlation between RNA integrity and rt-PCR experiments**. The figure shows the correlation between RNA integrity values and the outcome of an real-time-PCR experiment, i.e. the average expression values of 4 housekeeping genes (GAPDH, KYNF, NEFL, *β*2M). The vertical line is a meaningful threshold value for RIN classification, while the horizontal separates acceptable from unacceptable real-time PCR results, a) The RIN shows a strong correlation (0.52) to the expression value of the house keeping genes. A straightforward separation into *positives *and *negatives *is possible. b) The ribosomal ratio shows a poor correlation (0.24) to the expression value of the house keeping genes.

In the present study, RIN values as well as ribosomal ratios were correlated with real-time PCR data. A detailed description of the sample types and extraction methods as well of the entire experimental setup has been published previously [5]. In short, a gene score was calculated based on the average apparent expression level of 4 different housekeeping genes (GAPDH, KYNF, NEFL, *β*2M) as measured by real-time PCR. Please note that in this experiment, differences in the apparent gene expression levels are induced by progressing degradation of the RNA string material. Figure 6 shows the plot of the average apparent gene expression on a logarithmic scale against the RIN. Immediately 2 cluster of data appear corresponding to high gene expression (intact RNA) with a high RIN value and low gene expression (degraded RNA) with a low RIN value. On the other hand, the ribosomal ratio exhibits only a weak correlation with the experimentally observed gene expression level (RNA integrity). The RIN allows for a straightforward separation in *positives *and *negatives*, whereas the ribosomal ratio would reject many more experiments than necessary. The historical value of 2.0 would reject about 40 experiments of good quality and a value of 1.75 results in about 15 false negatives.

## Discussion

Because of the critical influence of RNA integrity on downstream experiments, there is a strong need for a reliable, reproducible, and standardized approach to classify the quality of RNA samples. The long time standard consisting in a 28S to 18S peak ratio of 2.0 was shown to provide only weak correlation with RNA integrity.

The Agilent 2100 bioanalyzer, a bio-analytical device based on a combination of microfluidics, microcapillary electrophoresis, and fluorescence detection, provides a platform to record the size distribution of molecules, e.g., RNA, in a digital format. Since this approach is highly reproducible and automated, it provides the basis for an automated, user-independent, and reproducible approach to evaluate the integrity of RNA samples using a software algorithm.

For the development of the RNA Integrity Number algorithm, a total of 1208 RNA samples from various sources and of different degradation states was analyzed. After assigning the samples to 10 different categories ranging from 1 (worst) to 10 (best), methods from information theory were applied to calculate features describing the curve of the electropherogram. In the following step, features were selected for further processing that showed high information content about the task to distinguish the 10 categories. These features were then taken as input variables for a model-training step. Here, using a Bayesian learning approach to select the most probable model, several models were trained utilizing artificial neural networks and the best was chosen for prediction of previously unseen test data. The result produced by this procedure is an algorithm called RNA Integrity Number (RIN).

### Analysis of the RIN model

The RIN algorithm is based on a selection of features that contribute information about the RNA integrity. It is obvious, that a single feature is hardly sufficient for a universal integrity measure. Moreover, a combination of different features covers several aspects of the measurement and is more robust against noise in the signal (see Additional file 2 for a overview of all features). To understand why the features were selected and to enhance the confidence for application specialists it is important, to give an interpretation of the features:

1. The total RNA ratio measures the fraction of the area in the region of 18S and 28S compared to the total area under the curve and reflects the proportion of large molecules compared to smaller ones. It has large values for categories 6 to 10.

2. The height of the 28S peak contributes additional information about the state of the degradation process, i.e. during degradation, the 28S band disappears faster than the 18S band. Therefore, it allows detection of a beginning degradation. It has largest values for categories 9 and 10, and zero values for categories 1 to 3.

3. The fast area ratio reflects how far the degradation proceeded and has typically larger values for the categories 3 to 6.

4. The marker height has large values for categories 1 and 2 and small values for all other categories since short degradation products will overlap with the lower marker.

**Figure 7:**
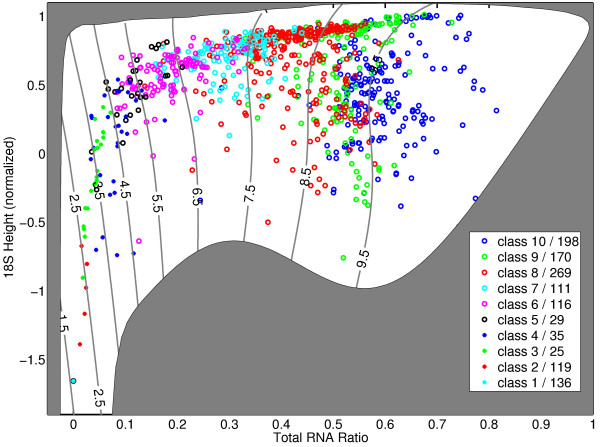
**2D visualization of integrity categories**. The figure shows a projection of the categories onto the two-dimensional space spanned by the first two features of the selected combination. These are total RNA ratio and 28S peak height. The experiments are clearly grouped along a curve from the left bottom corner up to the top and then to the right top corner. The variance in location of the experiments increases with larger categorical value. Categories 1 and 2 have almost no variance in this feature space. The grey border in the domain is given by the abnormality detectors for this two variables, i.e., for a data point outside the white area no RIN is computed.

Figure 7 shows the projection of the distribution of integrity categories onto a two-dimensional space spanned by the two most important features. Clearly, a global non-linear relationship can be observed. The experiments are grouped along a characteristic line with varying variance. The boundaries between adjacent categories are not perfectly sharp, but clearly visible in this projection with some interchanges.

### Comparing the approaches

Using a single simple feature to judge RNA Integrity was already shown to be insufficient [2,5]. While focusing on one aspect of the electropherogram allows for a rough orientation about the integrity, it is still subjective to a high degree. Linear models based on these features show a mean squared error that is four to sixty times higher (degradation factor resp. 28S/18S ratio) than compared to the proposed approach.

The reason for this tremendous difference lies in the fact, that neither the 28S/18S ratio nor the degradation factor reflect all properties of the RNA degradation process. For example, several samples of integrity category 10 are labeled *BLACK *from the degradometer software as they have low signal intensities. This happened for 42% of the samples under consideration, which are all samples that were under investigation for microarray experiments. The degradation factor contains similar information as the fast area ratio, which reflects typical characteristics of categories 3 to 7. The high ribosomal ratio is useful to detect a certain amount of high quality samples, but the categorization is not valid for all of them. Using several features which complement one another and allow for a non-linear weighting of these features allowed to reduce the error to a minimum value which is in the order of the natural noise in the target data. The noise results from using a categorical grid for a continues process as well as from a few abnormalities. Interestingly, almost no interchanges over more than one categorical border are observed. Thus, the classification errors appear almost only at the borderline between two categories, which was also difficult for humans to decide, when labeling the data.

### Availability of the RIN model

The Agilent 2100 bioanalyzer system software can be downloaded from Agilent's webpage [see Additional file 1]. Version B.01.03 and later will allow for measurement reviews (free of licenses) including the calculation of the RNA integrity number [8]. Up-to-date information about the RIN-project can be found at the RIN web site [9].

## Conclusion

This article investigates an automated procedure for extracting features from signal measurements, selecting suitable subsets of features and extracting a functional relationship to target labels of experiments. We demonstrated that the application of the methodology to a large collection of electrophoretic RNA measurements recorded with an Agilent 2100 bioanalyzer provides a predictive model for RNA integrity, the RIN. The generalization error is as low as the natural noise in the target values and apparently lower than for ribosomal ratios.

Our results show the importance of taking characteristics of several regions of the recorded electropherogram into account in order to get a robust and reliable prediction of RNA integrity, Furthermore, it was demonstrated that the RIN can be correlated with the outcome of downstream experiments, i.e. by performing this quality assessment step users can prevent themselves from erroneous results and loss of money and resources.

We conclude, that the RNA integrity number is an important tool in conducting valid gene expression measurement experiments as real-time PCR or DNA microarray, that is already widely and successfully used since the release of the *β*-version. It is largely free from instrument and concentration variability, thereby facilitating the comparison of samples between different labs. For example, the RIN can be assigned to a RNA sample before shipping from a lab and the same quality control can be performed in a user-independent way at the destination lab.

## Methods

### Data labeling and preprocessing

For classification of RNA integrity, ten categories were defined from 1 (totally degraded RNA) to 10 (fully intact RNA). Figure 2 illustrates qualitatively the differences between the categories. Each of the 1208 samples was assigned manually to one of the categories by experienced expert users.

The categorical values provide the target values for the adaptive learning steps. The assignment to categories was very carefully done as it is critical for the performance of the resulting algorithm. Especially for RNA samples at the borderline of two adjacent integrity categories, the assignment to each of the two categories could be justified but one had to be selected. This reflects a natural randomness which is inherent in a gradual process like RNA degradation. However, such random noise in the target values can easily be handled by the learning model, which assumes noise in the target data.

Detecting abnormalities in the electropherogram is another important preprocessing step to get a clean set of training samples. Various anomalies can disturb the usual shape of an electropherogram, e.g., ghost peaks, spikes, wavy baseline, and unexpected sample type. They were observed in approx. 5% of the samples. To separate anomalies from normal samples, several simple detectors were constructed. Each detector performs a linear classification based on a threshold value. Spikes, for example, can have a large peak height but have very narrow peak width; they appear as very sharp peaks. Normally, the largest peaks in the electrophoretic traces are located at the 18S and 28S bands but compared to a spike are significantly broader. If a high peak does not cover the minimal requested area, it is rejected as a spike and marked as abnormal. Applying these detection criteria to the data set returned 117 electropherograms as abnormal. Eleven abnormal samples could not be detected for example, because a spike arose near the 28S peak and could not be identified as such. All of them were assigned a sensible label and put in the test set. This reflects the natural occurrence of such effects in the test phase.

**Figure 8:**
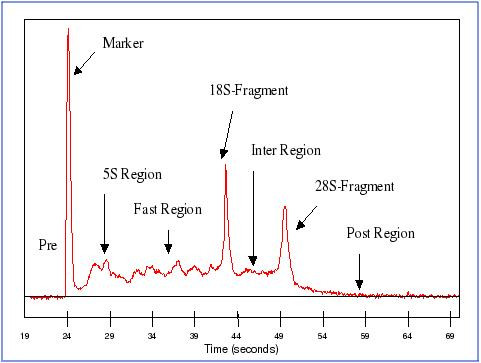
**Feature extraction**. Segments of an electropherogram: The segment preceeding the lower marker is designated the pre-region. The marker-region coincides with the area occupied by the lower-marker peak. The 5S-region covers the small rRNA fragments (5S and 5.8S rRNA, and tRNA). The 18S-region and 28S-region cover the 18S peak and 28S peak, respectively. The fast-region lies between the 5S-region and the 18S-region. The inter-region lies between the 18S-region and the 28S-region. The precursor-region covers the pre-cursor RNA following the 28S-region. And finally the post-region lies beyond the precursor-region.

In the application phase we distinguish between critical and non-critical anomalies based on their influence on the computation of the RIN. The former are anomalies of baseline and anomalies in the 5S-region, the latter anomalies in the pre-region, precursor-region and post-region (cf. fig. 8).

If a critical anomaly is detected, the RIN is not computed. Instead, an error message appears to the user. If a non-critical anomaly is detected, the RIN is computed and a warning to the user is displayed [10,11]. Baseline correction and normalization are applied to the electropherogram prior to the actual feature extraction process. These functions are standard features of Agilent's Expert Software [12]. The baseline is a constant background signal of the electropherogram and its level may significantly differ between different electropherograms. The baseline-corrected signal is then normalized. For height related features it is normalized to the global maximum of the 5S-region to precursor-region. For area related features it is normalized to the global signal area in the 5S-region to precursor-region. The pre-region, marker-region and post-region are intentionally not considered critical elements of the electrophoretic trace since they don't contain critical information about the RNA degradation process.

### Feature extraction

The aim of this step is to define and extract informative features from the electropherograms. For this purpose, each electropherogram is divided into the following nine adjacent segments covering the entire electropherogram: a pre-region, a marker-region, a 5S-region, a fast-region, an 18S-region, an inter-region, a 28S-region, a precursor-region and a post-region (Fig. 8). The subdivision is based on the peak table and the fragment table as computed by Agilent's Expert Software.

Each of these segments can then be considered separately yielding a number of local features. Several iterations of generating and evaluating features showed that it is sufficient to extract a set of specific features reflecting statistical properties, such as average and maximum heights, areas and their ratios as well as signal-to-noise ratios to cover for the information contained in the different regions of an electropherogram.

In addition, several global features were extracted, i.e. features that span several segments. Among these, the average and maximum height, areas and their ratios, total RNA ratio and the 28S area ratio are the most important features. Both have been used as criteria for RNA integrity assessment in the past. The total RNA ratio is the ratio of the area of the ribosomal bands to the total area of the electropherogram, whereas 28S area ratio measures only the fraction of 28S-fragment. This set of features extracted from the electropherograms and their manually assigned RNA integrity categories form the knowledge base for the following steps of the algorithm. Additional file 2 contains a complete description of the set of features.

### Feature selection

In practice, only a limited amount of data is available for determining the parameters of a model. Therefore, the dimensionality of the feature vector must be in a sensible relation to the number of data points, i.e. electropherograms. Each additional feature increases the information content of the input about the target, but decreases the number of data points per dimension in order to determine the parameters. This means that the class of functions in which the solution is searched increases fast ('empty space phenomenon') [13]. Estimating a dependency between the features and the target will be more robust when the input-target space is low-dimensional. A rough orientation to determine the maximum number of features for training a prediction model is given in [14]. For a data set of about 1000 experiments the search is therefore restricted to vectors which combine at most eight features. To select the most promising features from our candidates, we use a forward selection procedure based on mutual information. The mutual information *MI *(*X *; T) of two random vectors *X *and *T *is defined as the Kullback-Leibler distance between the joint distribution *p*(*x*, *t*) and the product *p*(*x*)*p*(*t*):

MI(X;T)=∫∫p(x,t)⋅log⁡p(x,t)p(x)p(t)dx dt     (1)
 MathType@MTEF@5@5@+=feaafiart1ev1aaatCvAUfKttLearuWrP9MDH5MBPbIqV92AaeXatLxBI9gBaebbnrfifHhDYfgasaacH8akY=wiFfYdH8Gipec8Eeeu0xXdbba9frFj0=OqFfea0dXdd9vqai=hGuQ8kuc9pgc9s8qqaq=dirpe0xb9q8qiLsFr0=vr0=vr0dc8meaabaqaciaacaGaaeqabaqabeGadaaakeaacqWGnbqtcqWGjbqscqGGOaakcqWGybawcqGG7aWocqWGubavcqGGPaqkcqGH9aqpdaWdbaqaamaapeaabaGaemiCaaNaeiikaGIaemiEaGNaeiilaWIaemiDaqNaeiykaKIaeyyXICTagiiBaWMaei4Ba8Maei4zaC2aaSaaaeaacqWGWbaCcqGGOaakcqWG4baEcqGGSaalcqWG0baDcqGGPaqkaeaacqWGWbaCcqGGOaakcqWG4baEcqGGPaqkcqWGWbaCcqGGOaakcqWG0baDcqGGPaqkaaGaemizaqMaemiEaGNaaGPaVlabdsgaKjabdsha0jaaxMaacaWLjaWaaeWaaeaacqaIXaqmaiaawIcacaGLPaaaaSqabeqaniabgUIiYdaaleqabeqdcqGHRiI8aaaa@6136@

and measures the degree of stochastic dependency between the two random vectors [15]. This can be expressed in terms of entropies as

*MI *(*X*; *T*) = *H*(*T*) - *H*(*T*|*X*)     (2)

which describes the reduction in the uncertainty or entropy *H*(*T*) of a target vector *T *due to the knowledge of *X. *To compute the required densities in equation 1 from the empirical data, we use non-parametric kernel estimators as described in [14,16].

As the first step in the selection procedure, a feature *f *is selected from the set of candidates which has maximal mutual information with the target for the given data set. In each subsequent step, one of the remaining features is selected which maximizes the mutual information with the target when added to the already selected features. This way the features are ranked by the amount of information they add about the target.

The result of the feature selection process is a sorted list (cf. table 2 which allows constructing a nested series of feature spaces with growing dimensionality up to the maximal amount of *d = *8 features:

{*f*_1_} ⊂ {*f*_1_, *f*_2_} ⊂ {*f*_1_, *f*_2_, *f*_3_} ⊂ ... ⊂ {*f*_1_,..., *f*_8_}

### Model training

After the nested series is constructed, regression models are trained for each feature space. Neural networks are used as semi-parametric regression models [16,17].

The functional relationship between the input features and the target, which has to be learned from the empirical data, must generalize to previously unseen data points, i.e. electropherograms of newly measured RNA. The theory of regularization shows that approximating the target as good as possible on the training data, for example, by minimizing the mean squared error *E*_*D*_, is not sufficient: it is crucial to balance the training error against the model complexity [17]. Therefore, we train the neural networks to minimize the regularized error *E = βED + αE*_*R*_. The regularization term *E*_*r *_measures the model complexity, taking into account the weights *w*_*k *_in the network. We choose the weight-decay 12∑wk2
 MathType@MTEF@5@5@+=feaafiart1ev1aaatCvAUfKttLearuWrP9MDH5MBPbIqV92AaeXatLxBI9gBaebbnrfifHhDYfgasaacH8akY=wiFfYdH8Gipec8Eeeu0xXdbba9frFj0=OqFfea0dXdd9vqai=hGuQ8kuc9pgc9s8qqaq=dirpe0xb9q8qiLsFr0=vr0=vr0dc8meaabaqaciaacaGaaeqabaqabeGadaaakeaadaWcaaqaaiabigdaXaqaaiabikdaYaaadaaeabqaaiabdEha3naaBaaaleaacqWGRbWAdaahaaadbeqaaiabikdaYaaaaSqabaaabeqab0GaeyyeIuoaaaa@34D4@ as the regularization term. The factors *α *and *β *are additional control parameters, i.e. hyperparameters.

We apply a Bayesian approach to determine the weights *w*_*k*_, and the parameters *α *and *β *during training as described elsewhere [16,18]. Given the training data *D *we want to find the 'best' model *M*(Θ) with parameter vector Θ. This is expressed in Bayes' theorem as follows

p(Θ|D)=p(D|Θ)p(Θ)p(D)     (3)
 MathType@MTEF@5@5@+=feaafiart1ev1aaatCvAUfKttLearuWrP9MDH5MBPbIqV92AaeXatLxBI9gBaebbnrfifHhDYfgasaacH8akY=wiFfYdH8Gipec8Eeeu0xXdbba9frFj0=OqFfea0dXdd9vqai=hGuQ8kuc9pgc9s8qqaq=dirpe0xb9q8qiLsFr0=vr0=vr0dc8meaabaqaciaacaGaaeqabaqabeGadaaakeaacqWGWbaCcqGGOaakcqqHyoqucqGG8baFcqWGebarcqGGPaqkcqGH9aqpdaWcaaqaaiabdchaWjabcIcaOiabdseaejabcYha8jabfI5arjabcMcaPiabdchaWjabcIcaOiabfI5arjabcMcaPaqaaiabdchaWjabcIcaOiabdseaejabcMcaPaaacaWLjaGaaCzcamaabmaabaGaeG4mamdacaGLOaGaayzkaaaaaa@4887@

The best model maximizes the posterior probability, i.e. we want to find Θ* such that *p*(Θ*|*D*) ≥ *p*(Θ|*D*) ∀ Θ.

In case of neural networks, the parameter vector consists of the weight vector **w**, the hyperparameters *α *and *β *as well as the topology of the network. In general, we cannot determine all these parameters at once.

For that, the Bayesian framework proceeds in a hierarchical fashion. While keeping all other parameters fixed on the first level, we search for an optimal weight vector which maximizes the posterior probability *p*(**w**|D) using the fast optimization algorithm Rprop [19].

On the second level, optimal weighting coefficients *α *and *β *are determined which again maximize the posterior probability *p*(*α,β*|*D*). This is done iteratively: we fix *α *and *β *and optimize the weights *w*_*k *_as described above. Afterwards, we re-estimate *α *and *β *and keep alternating several times between optimizing the weights and updating *α *and *β *(see [16] for the update rules and mathematical details).

On the third level, we can compare networks with different topologies. Using Bayes' rule we can write the posterior probability of a model ℋ
 MathType@MTEF@5@5@+=feaafiart1ev1aaatCvAUfKttLearuWrP9MDH5MBPbIqV92AaeXatLxBI9gBamrtHrhAL1wy0L2yHvtyaeHbnfgDOvwBHrxAJfwnaebbnrfifHhDYfgasaacH8akY=wiFfYdH8Gipec8Eeeu0xXdbba9frFj0=OqFfea0dXdd9vqai=hGuQ8kuc9pgc9s8qqaq=dirpe0xb9q8qiLsFr0=vr0=vr0dc8meaabaqaciaacaGaaeqabaWaaeGaeaaakeaaimaacqWFlecsaaa@3763@ as *P*(ℋ
 MathType@MTEF@5@5@+=feaafiart1ev1aaatCvAUfKttLearuWrP9MDH5MBPbIqV92AaeXatLxBI9gBamrtHrhAL1wy0L2yHvtyaeHbnfgDOvwBHrxAJfwnaebbnrfifHhDYfgasaacH8akY=wiFfYdH8Gipec8Eeeu0xXdbba9frFj0=OqFfea0dXdd9vqai=hGuQ8kuc9pgc9s8qqaq=dirpe0xb9q8qiLsFr0=vr0=vr0dc8meaabaqaciaacaGaaeqabaWaaeGaeaaakeaaimaacqWFlecsaaa@3763@|*D*) = *p*(*D*|ℋ
 MathType@MTEF@5@5@+=feaafiart1ev1aaatCvAUfKttLearuWrP9MDH5MBPbIqV92AaeXatLxBI9gBamrtHrhAL1wy0L2yHvtyaeHbnfgDOvwBHrxAJfwnaebbnrfifHhDYfgasaacH8akY=wiFfYdH8Gipec8Eeeu0xXdbba9frFj0=OqFfea0dXdd9vqai=hGuQ8kuc9pgc9s8qqaq=dirpe0xb9q8qiLsFr0=vr0=vr0dc8meaabaqaciaacaGaaeqabaWaaeGaeaaakeaaimaacqWFlecsaaa@3763@)·*P*(ℋ
 MathType@MTEF@5@5@+=feaafiart1ev1aaatCvAUfKttLearuWrP9MDH5MBPbIqV92AaeXatLxBI9gBamrtHrhAL1wy0L2yHvtyaeHbnfgDOvwBHrxAJfwnaebbnrfifHhDYfgasaacH8akY=wiFfYdH8Gipec8Eeeu0xXdbba9frFj0=OqFfea0dXdd9vqai=hGuQ8kuc9pgc9s8qqaq=dirpe0xb9q8qiLsFr0=vr0=vr0dc8meaabaqaciaacaGaaeqabaWaaeGaeaaakeaaimaacqWFlecsaaa@3763@)/*p*(*D*). If we assign the same prior *P*(ℋ
 MathType@MTEF@5@5@+=feaafiart1ev1aaatCvAUfKttLearuWrP9MDH5MBPbIqV92AaeXatLxBI9gBamrtHrhAL1wy0L2yHvtyaeHbnfgDOvwBHrxAJfwnaebbnrfifHhDYfgasaacH8akY=wiFfYdH8Gipec8Eeeu0xXdbba9frFj0=OqFfea0dXdd9vqai=hGuQ8kuc9pgc9s8qqaq=dirpe0xb9q8qiLsFr0=vr0=vr0dc8meaabaqaciaacaGaaeqabaWaaeGaeaaakeaaimaacqWFlecsaaa@3763@) to every model, it is sufficient to evaluate the quantity *p*(*D*|ℋ
 MathType@MTEF@5@5@+=feaafiart1ev1aaatCvAUfKttLearuWrP9MDH5MBPbIqV92AaeXatLxBI9gBamrtHrhAL1wy0L2yHvtyaeHbnfgDOvwBHrxAJfwnaebbnrfifHhDYfgasaacH8akY=wiFfYdH8Gipec8Eeeu0xXdbba9frFj0=OqFfea0dXdd9vqai=hGuQ8kuc9pgc9s8qqaq=dirpe0xb9q8qiLsFr0=vr0=vr0dc8meaabaqaciaacaGaaeqabaWaaeGaeaaakeaaimaacqWFlecsaaa@3763@), which is called the evidence for ℋ
 MathType@MTEF@5@5@+=feaafiart1ev1aaatCvAUfKttLearuWrP9MDH5MBPbIqV92AaeXatLxBI9gBamrtHrhAL1wy0L2yHvtyaeHbnfgDOvwBHrxAJfwnaebbnrfifHhDYfgasaacH8akY=wiFfYdH8Gipec8Eeeu0xXdbba9frFj0=OqFfea0dXdd9vqai=hGuQ8kuc9pgc9s8qqaq=dirpe0xb9q8qiLsFr0=vr0=vr0dc8meaabaqaciaacaGaaeqabaWaaeGaeaaakeaaimaacqWFlecsaaa@3763@[17,18]. Marginalizing over *α *and *β *and making use of the posterior probability for *α *and *β *from the second level provides an equation for the logarithm of the evidence ln *p*(*D*|ℋ
 MathType@MTEF@5@5@+=feaafiart1ev1aaatCvAUfKttLearuWrP9MDH5MBPbIqV92AaeXatLxBI9gBamrtHrhAL1wy0L2yHvtyaeHbnfgDOvwBHrxAJfwnaebbnrfifHhDYfgasaacH8akY=wiFfYdH8Gipec8Eeeu0xXdbba9frFj0=OqFfea0dXdd9vqai=hGuQ8kuc9pgc9s8qqaq=dirpe0xb9q8qiLsFr0=vr0=vr0dc8meaabaqaciaacaGaaeqabaWaaeGaeaaakeaaimaacqWFlecsaaa@3763@), which depends on the training error, the possible symmetries resulting from the network topology, the size of the hyperparameters *α *and *β*, the number of samples and the number of weights, the second derivative of the error (Hessian matrix) respective its determinant, as well as the eigenvalues of the Hessian, thus reflecting the balance between these terms (cf. [16]).

To find the model which best explains the given data set i.e. with maximal evidence, we systematically vary the number *h *of hidden units in the networks from 0 to 8. All hidden units with *tangens hyperbolicus *as activation function are in a single layer, restricting the search space to models with moderate non-linearity. The best topology is selected as the topology with the highest evidence in average over a 10-fold cross-validation procedure on the training data. This topology is then used to train the final model on the whole training set. Figure 6 shows a strong correlation between the logarithm of the model evidence and the cross-validation error, pointing out that the evidence is a sensible selection criterion.

## Authors' contributions

AS, OM, SS, RS, ML and TR designed the study. AS, OM, SS, RS, ML, MGA, SL, WM, MGR and TR contributed to development of methodology. AS and TR executed the experiments, AS, OM, SS, RS, SL and MGA validated the study. AS, MGR and TR designed and implemented the software for this study. Illustrations from AS, OM, MGR and TR. All authors participated in writing the manuscript. All authors approved the final manuscript.

## Supplementary Material

Additional File 2 Description of the total set of features computed Click here for file

Additional File 1 Software availability Click here for file

## Figures and Tables

**Table 1:** Description of the data. The table summarizes the distribution of electropherograms over categories and the percentages for training and test sets are given.

Category	Training set	in %	Test set	in %	Total	in %
1	190	20.3%	77	19.2%	267	20.0%
2	84	9.0%	35	8.7%	119	8.9%
3	17	1.8%	8	2,0%	25	1.9%
4	27	2.9%	8	2.0%	35	2.6%
5	17	1.8%	12	3.0%	29	2.2%
6	81	8.6%	35	8.7%	116	8.7%
7	80	8.5%	31	7.7%	111	8.3%
8	188	20.1%	81	20.1%	269	20.1%
9	117	12.5%	53	13.2%	170	12.7%
10	136	14.5%	62	15.4%	198	14.8%

Total	937		402		1339	

**Table 2:** Iterative feature ranking. The table gives the results of the iterative forward search. The 2nd column shows the percentage of information in the input vector compared to the entropy of the target. The 3rd column gives the values for single features and the 4th column the rank of the single features among all features. Feature names [see Additional file 2]: 1. total.rna.ratio 2. fr28s.height 3. fr28s.area.ratio 4. ratio1828.fast 5. reg5s.endval 6. fast.area.ratio 7. fr18s.found 8. all.mean.median

Step	*MI*(*X,T*)/*H*(*T*) (Combination)	*MI*(*X*_*i*_,*T*)/*H*(*T*) (single feature)	Rank of single feature
1	0.79	0.79	1
2	0.79	0.50	38
3	0.81	0.74	3
4	0.83	0.55	19
5	0.83	0.46	51
6	0.85	0.60	10
7	0.85	0.47	48
8	0.86	0.50	39

**Table 3:** Comparison of model results. The table summarizes the results for three different models on a subset of 439 samples. The amount of information provided by the features is compared as well as the model evidences and the cross validation error. The values in parentheses give the results for a reduced sample set, which contains no samples labeled *BLACK *with the degradometer software.

	28S/18S ratio	degradation factor	RIN model
MI (X;T)/H(T)		0.43 (0.63)	0.88
log evidence	-1457	-577 (-121)	+3.3
MSE (CV)	6.3	2.83 (0.60)	0.15

**Table 4:** Proportion of misclassified samples. The table shows an detailed analysis of sample assignment to integrity categories. Each category *c*_*k *_is tested against a series of set union ∪*c*_*i *_of all other categories *i *with *i *= 1,...,*k *- 1. The RIN of each electropherogram is rounded up or down to the closest categorical value. The classification performance for assigning electropherograms to the two resulting sets is then measured by Receiver Operating Characteristics, more precisely by the area under the curve (cf. fig. 5). There are always a few interchanges between adjacent categories, but rarely interchanges over two categorical borders. Values are rounded to two decimals.

	9	8	7	6	5	4	3	2	1
10	0.96	0.98	1.00	1.00	1.00	1.00	1.00	1.00	1.00
9		0.96	1.00	1.00	1.00	1.00	1.00	1.00	1.00
8			0.98	0.99	1.00	1.00	1.00	1.00	0.99
7				0.98	0.99	0.99	0.99	0.99	0.98
6					0.96	0.98	0.98	0.99	0.98
5						0.95	0.97	0.98	0.98
4							0.98	0.99	0.98
3								0.99	0.98
2									0.95
