# Supplementary material for: The RIN: an RNA integrity number for assigning integrity values to RNA measurements
Source: BMC Mol Biol. 2006 Jan 31;7:3. doi: 10.1186/1471-2199-7-3 (PMC1413964; doi:10.1186/1471-2199-7-3)
Supplement: Additional File 2 — Description of the total set of features computed [file 1471-2199-7-3-S2.pdf]

Description of the total set of features computed

Table 1: The table list the names of the regions and their definition

Name	Description
pre-region	The segment preceeding the lower marker is designated the pre-region.
marker-region	The area occupied by the lower-marker peak
5S-region	covers the small rRNA fragments (5S and 5.8S rRNA, and tRNA)
fast-region	lies between the 5S-region and the 18S-region
18S-region	covers the 18S peak
28S-region	covers 28S peak
inter-region	lies between the 18S-region and the 28S-region
precursor-region	covers the pre-cursor RNA following the 28S-region
post-region	part of the signal that lies beyond the precursor-region

Table 2: The table gives for each used feature a short description of its semantics

Feature	Description
**Fragment resp. marker features**	
fragmentarea	area of the fragment
area	area under the curve
found	fragment/marker recognized
height	height of a fragment/marker
max	max value of the fragment/marker
**Region features**	
area	area under the curve in the region
cuttedarea	area under the curve with peaks cutted off
abovecuttedarea	sum of the cutted off peak areas
line(1)	slope of the interpolated straight line
line(2)	intercept of the interpolated straight line
startval	value of the interpolated straight line at the beginning of the region
endval	value of the interpolated straight line at the end of the region
median	median value of the region
mean	mean value of the region
max	max value of the region
min	min value of the region
runmindeviation	max deviation of the running minimum and the current signal value
runmaxdeviation	max deviation of the running maximum and the current signal value.
arearatio	area ratio of the region and the total area under the curve
countintpeaks	number of peaks found by the integrator inside the region
meme	ratio of region's mean and median (is helpful for spike detection)
**Global features**	
ratio18_fast	area ratio of 18S fragment and fast region
ratio28_fast	area ratio of 28S fragment and fast region
ratio1828_fast	area ratio of both ribosomal fragments and fast region
ratio18_inter	area ratio of 18S fragment and inter region
ratio28_inter	area ratio of 28S fragment and inter region
ratio1828_inter	area ratio of both ribosomal fragments and inter region
ratio18_fastinter	area ratio of 18S fragment and both fast region and inter region
ratio28_fastinter	area ratio of 28S fragment and both fast region and inter region
ratio1828_fastinter	area ratio of both ribosomal fragments and fast + inter region
arearatio2818	area ratio of 28S fragment and 18S fragment
ratio18_fastmedian	area ratio of 18S fragment and median value of fast region - our counterpart to the degradation factor
totalrnaratio	area ratio of both ribosomal fragments to the total area under the curve (reaching from 5S region to the precursor region)
SNR	signal noise ratio
NSD	noise standard deviation
baseline(1)	slope of the baseline, put through the lowest points of the pre region and post region
baseline(2)	intercept of the baseline
