# Supplementary material for: The RIN: an RNA integrity number for assigning integrity values to RNA measurements
Source: BMC Mol Biol. 2006 Jan 31;7:3. doi: 10.1186/1471-2199-7-3 (PMC1413964; doi:10.1186/1471-2199-7-3)
Supplement: Additional File 1 — Software availability [file 1471-2199-7-3-S1.pdf]

Software Availability

The Agilent 2100 bioanalyzer system software can be downloaded from Agilent's webpage. Version B.01.03 and later will allow for measurement reviews (free of licenses) including the calculation of the RNA integrity number: http://www.agilent.com/chem/labonachip

Up-to-date information for the RIN Software and about the RIN-Project is available at

http://www.agilent.com/chem/RIN and http://www.quantiom.com/RIN
